# Differentiate preterm and term infant brains and characterize the corresponding biomarkers *via* DICCCOL-based multi-modality graph neural networks

**DOI:** 10.3389/fnins.2022.951508

**Published:** 2022-10-14

**Authors:** Shu Zhang, Ruoyang Wang, Junxin Wang, Zhibin He, Jinru Wu, Yanqing Kang, Yin Zhang, Huan Gao, Xintao Hu, Tuo Zhang

**Affiliations:** ^1^Center for Brain and Brain-Inspired Computing Research, School of Computer Science, Northwestern Polytechnical University, Xi'an, China; ^2^School of Automation, Northwestern Polytechnical University, Xi'an, China

**Keywords:** preterm infant brain, DICCCOL, multi-modality, GNN, biomarker

## Abstract

Preterm birth is a worldwide problem that affects infants throughout their lives significantly. Therefore, differentiating brain disorders, and further identifying and characterizing the corresponding biomarkers are key issues to investigate the effects of preterm birth, which facilitates the interventions for neuroprotection and improves outcomes of prematurity. Until now, many efforts have been made to study the effects of preterm birth; however, most of the studies merely focus on either functional or structural perspective. In addition, an effective framework not only jointly studies the brain function and structure at a group-level, but also retains the individual differences among the subjects. In this study, a novel dense individualized and common connectivity-based cortical landmarks (DICCCOL)-based multi-modality graph neural networks (DM-GNN) framework is proposed to differentiate preterm and term infant brains and characterize the corresponding biomarkers. This framework adopts the DICCCOL system as the initialized graph node of GNN for each subject, utilizing both functional and structural profiles and effectively retaining the individual differences. To be specific, functional magnetic resonance imaging (fMRI) of the brain provides the features for the graph nodes, and brain fiber connectivity is utilized as the structural representation of the graph edges. Self-attention graph pooling (SAGPOOL)-based GNN is then applied to jointly study the function and structure of the brain and identify the biomarkers. Our results successfully demonstrate that the proposed framework can effectively differentiate the preterm and term infant brains. Furthermore, the self-attention-based mechanism can accurately calculate the attention score and recognize the most significant biomarkers. In this study, not only 87.6% classification accuracy is observed for the developing Human Connectome Project (dHCP) dataset, but also distinguishing features are explored and extracted. Our study provides a novel and uniform framework to differentiate brain disorders and characterize the corresponding biomarkers.

## Introduction

Preterm birth is a worldwide problem that affects infants throughout their lives significantly. Fifteen million babies are estimated to be born prematurely each year, and ~1 million children die each year due to complications of preterm birth. The lifetime of disability, including learning disabilities, visual problems, and hearing problems will last in many survivors (Vogel et al., 2005; Douglas-Escobar and Weiss, 2013; Cecatti et al., 2016; Sheinerman et al., 2017; Walani, 2020). Furthermore, much evidence has been found for the fact that all aspects of brain development can be affected by preterm delivery (Berger et al., 2012). Therefore, it is urgent to further elucidate the differences between preterm and term infant brains, which will facilitate the interventions for neuroprotection and improve outcomes of prematurity.

To elucidate the abnormalities of the preterm infant brain structures, many investigations have been proposed to study the alterations on the cortical, white matter (WM), gray matter (GM), and deep GM volumes in preterm infant brains at the macro-structural level. With the assistance of advanced neuroimaging technologies, studies are proposed to characterize the alterations at the connectome-level from the functional or the structural perspective (Damaraju et al., 2010; Smyser et al., 2013; Wehrle et al., 2018). A few studies revealed that structural and functional alterations were found in preterm infant brains predominantly in frontal, temporal, and occipital regions, and in the cerebellum (Eikenes et al., 2011; Bjuland et al., 2014; Nosarti et al., 2014), which represented a greater potential of exploring their correlations. Some studies further elucidated that most structural-based alterations were more inclined to decrease the intensity of connectivity while the functional-based alterations owned the opposite situations. However, some other findings suggested that structural and functional perspectives are quite complex to associate, and structural and functional alterations are not always consistent (Kelly et al., 2019; Saha et al., 2020; Sa de Almeida et al., 2021). To further elucidate causes and relationships of functional and structural alterations, it is important to understand those alteration regions/connections that are directly or indirectly connected to structural and functional perspectives, realize whether they have common architecture, and further explore the possible hypothesis that functional alterations can be derived from the structural alterations. To achieve these goals, a study solely on single modality is not enough; multi-modality fusion is the key point. Therefore, the integration of the preterm infant brain, structural and functional connectivity profiles, and studying their relationship and exploring the distinguishing features/biomarkers are extremely important and necessary.

Advantages of the joint representation analysis are also obvious in the field of machine learning; it has been suggested that each different imaging technique should feedback different brain information (Sui et al., 2012). For example, functional magnetic resonance imaging (fMRI) measures the hemodynamic response related to neural activity in the brain dynamically; structural magnetic resonance imaging (sMRI) additionally provides information about structural connectivity among brain networks. Sui et al. (2012) believed that conjoint analysis could maximize the use of cross-information in the existing data, in finding important changes that are only partially detected in each modality. Some studies have dedicated efforts on the topic of multi-modality analysis (Qi et al., 2018; Sui et al., 2020). For example, a multimodal canonical correlation analysis model with joint independent component analysis was proposed (Sui et al., 2018). It was worth noting that in most existing methods, subjects were usually registered to a common atlas to align data across subjects (Chen et al., 2022). However, individual differences will be sacrificed in these approaches because image registration algorithms had difficulty in dealing with the anatomical variation that existed between different brains (Ardekani et al., 2004; Stiers et al., 2006; Thirion et al., 2006; Li et al., 2010, 2012).

In recent years, deep learning algorithms have shown the superiority of automatically studying and characterizing the distinguishing features from the medical images (Li et al., 2014; Suk et al., 2014; Litjens et al., 2017). Thus, the advanced learning algorithms are preferred to be adopted into the study of differentiating and characterizing brain diseases and biomarkers (Cole et al., 2017; Mehdipour Ghazi et al., 2019). Compared with convolutional neural network (CNN)- and recurrent neural networks (RNN)-based algorithms, graph neural networks (GNN)-based algorithms (Li et al., 2019, 2021) perform the convolution operations on the non-Euclidean data, which is more suitable to study the topological information of the brain networks and help identify the biomarkers from the high-dimensional functional and structural representations of the brain. Among existing GNN models, the self-attention graph pooling (SAGPOOL) (Lee et al., 2019) model is an effective model structure with fast training speed and is suitable for small datasets. It uses a three-layer graph convolution layer to extract features and a self-attention pooling layer to select important dense individualized and common connectivity-based cortical landmarks (DICCCOL) landmarks by updating their corresponding weights. This fits well with the purpose of classifying and analyzing relevant preterm and term biomarkers.

To effectively capture the biomarkers of brain disorders and diseases, e.g., disorders from preterm birth, from multi-modality and retain the individual differences, in this study, a novel DICCCOL-based multi-modality GNN (DM-GNN) is proposed to differentiate the brain disorders and characterize the corresponding biomarkers. The major advantages of the proposed DM-GNN framework are three-folds: (1) The DICCCOL system is introduced to initialize the graph nodes with correspondence and retain the individual differences. (2) Multi-modality representations are included in this work. DICCCOL-based functional and structural profiles are extracted from the magnetic resonance imaging (MRI) of the brain, which includes the brain anatomy (from DICCCOL system), function, and structure representations. (3) The GNN framework with SAGPOOL is proposed to feedback the importance of each graph node such that distinguishing biomarkers can be identified.

Our experiment results successfully demonstrate the effectiveness of the proposed approach. About 86.4% preterm-term classification accuracy is achieved and 107 DICCCOL landmarks are recognized as the biomarkers of greater importance. Particularly, precentral, precuneus, superior frontal, superior parietal, supramarginal, isthmus insula, and postcentral regions are further identified as the biomarker regions, which are studied and discussed throughout the study. Our results shed new insights that biomarkers can be successfully identified by the integration of multi-modality representations. It is worth noting that our proposed DM-GNN approach is applicable to many other brain disorders and diseases, which can be easily transferred and applied.

The remainder of this article is structured as follows: in Section Methods, we introduce the DM-GNN framework, and the experiment design is described in detail; in Section Results, the results are presented according to the experiment design, and biomarkers are identified and discussed. In Section Conclusion and discussion, the conclusion is drawn, and future perspective is also discussed.

## Methods

### Dataset and preprocessing

Eighty-six infant subjects are selected from the dataset of developing Human Connectome Project (dHCP) (Hughes et al., 2017; Makropoulos et al., 2018). Of these, 43 are preterm infants born < 38 weeks, and the remainder are term infants over 38 weeks. To compare the growth of preterm and term infants in different settings, we use data from term and preterm infants at the same gestational age. All the subjects are scanned with sMRI, diffusion MRI (dMRI), and resting-state fMRI (rs-fMRI) at around 40 weeks.

The basic parameters of T2-weighted sMRI are as follows: TR = 1,200 *ms*, TE = 156 *ms*, SENSE factor = 2.11 (axial) and 2.60 (sagittal), image matrix = 290 × 290 × 203, and resolution = 0.5 × 0.5 × 0.5 *mm*. Diffusion weighted images consist of three shells of b = 400, 1,000, and 2,600 *s*/*mm*^2^ and were interspersed with an approximately equal number of acquisitions on each shell within each run. The basic parameters of rs-fMRI are as follows: TR = 392 *ms*, TE = 38 *ms*, total volume = 2,300, image matrix = 67 × 67 × 45, and resolution = 2.16 × 2.16 × 2.15 *mm*. The basic parameters of dMRI are as follows: TR = 3,800 *ms*, TE = 90 *ms*, total slice = 300, SENSE factor = 1.2, partial Fourier = 0.86, image matrix = 128 × 128 × 64, and resolution = 1.17 × 1.17 × 1.5 *mm*. A spherically optimized set of directions on 4 shells (b0: 20, b400: 64, b1000: 88, b2600: 128) is split into four optimal subsets (one per phase encoding direction).

FMRIB Software Library (FSL) FEAT (Jenkinson et al., 2012) is adopted to process rs-fMRI data as follows: skull removal, motion correction, slice time correction, and spatial smoothing. It is worth noting that dMRI is used as an intra-subject standard space, to which the other data modalities are aligned. T2-weighted sMRI volumes are linearly warped to fractional anisotropy (FA) map of dMRI. Then, the surface is transposed to dMRI space by applying the transformation matrix onto it. We reconstruct the cortical surface using T2-weighted sMRI data, following the steps provided in the dHCP dataset: skull removal, tissue segmentation, and surface reconstruction. We process the dMRI data using the skull-strip and eddy current corrections of FSL (Jenkinson et al., 2012), and then use DSI Studio (Yeh, 2020) for fiber tracking.

### Proposed DICCCOL-based multi-modality GNN learning framework

**Figure 1:**
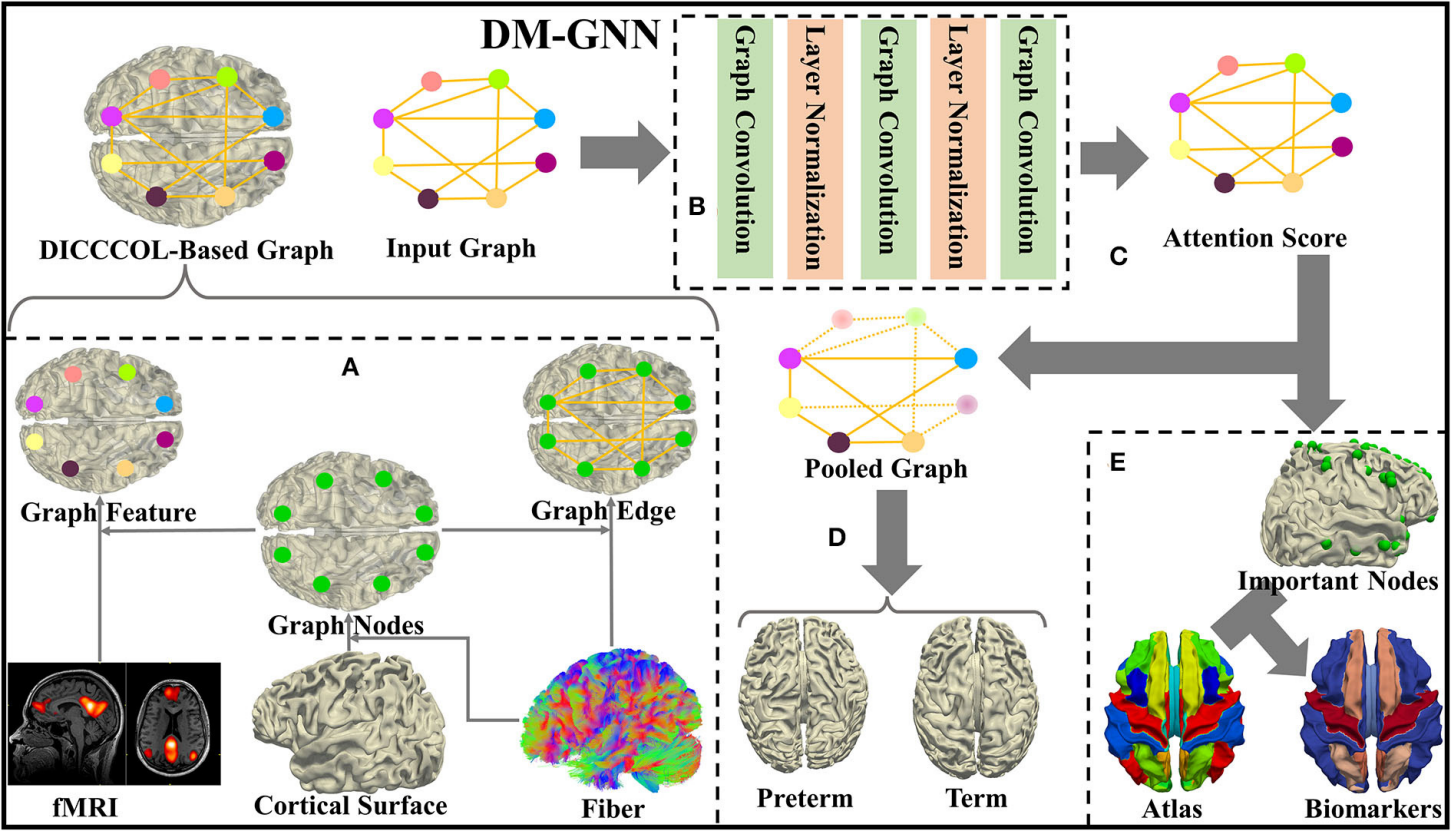
Framework of proposed dense individualized and common connectivity-based cortical landmarks (DICCCOL)-based multi-modality graph neural network (GNN). **(A)** Graph generation. **(B)** Feature extraction. **(C)** Graph pooling. **(D)** Classification. **(E)** Biomarker analysis.

In this study, a novel DM-GNN approach is proposed to differentiate the differences between the brains of preterm and term infants both at 40 weeks of gestational age and identify the corresponding biomarkers. The major steps can be summarized into five steps, i.e., graph generation, feature extraction, graph pooling, graph classification, and biomarker analysis. Please refer to Figure 1 for details. Specifically, in the graph generation module, we construct structural connectivity matrix and functional similarity matrix from dMRI, sMRI, and rs-fMRI data, and further represent these two matrices sparsely, which delegate edges and features of the graph, respectively. In the feature extraction module, we use three graph convolution layers to extract node features and concatenate the node features of the three layers together for the subsequent process. In the graph pooling module, we use the self-attention pooling to select important nodes by updating weights to nodes. In the graph classification module, we use global-maximum and global-average to aggregate all node features as features of the graph used for binary classification. In the model of our proposed DM-GNN framework, two parameters are vitally important. In the biomarker analysis module, we extract some DICCCOL landmarks as biomarkers and analyze the brain regions to which they belonged. They are number of layers and number of the hidden units, respectively. To explore the proper parameters, several experiments are applied using different parameters.

### Description of DICCCOL-based multi-modality GNN

In the DM-GNN model, first, we utilize DICCCOL landmarks to process multi-modality data into the input data of the model and use three graph convolutional layers to extract high-level features. Next, the features operated by the three graph convolution layers are spliced, and the attention score of those graph nodes is measured to reveal the contributions from each node for the task of differentiation. Finally, we input the features of DICCCOL landmarks with higher attention scores into two fully connected layers to classify preterm and term infants.

#### Graph generation

##### Generate nodes of graph

**Figure 2:**
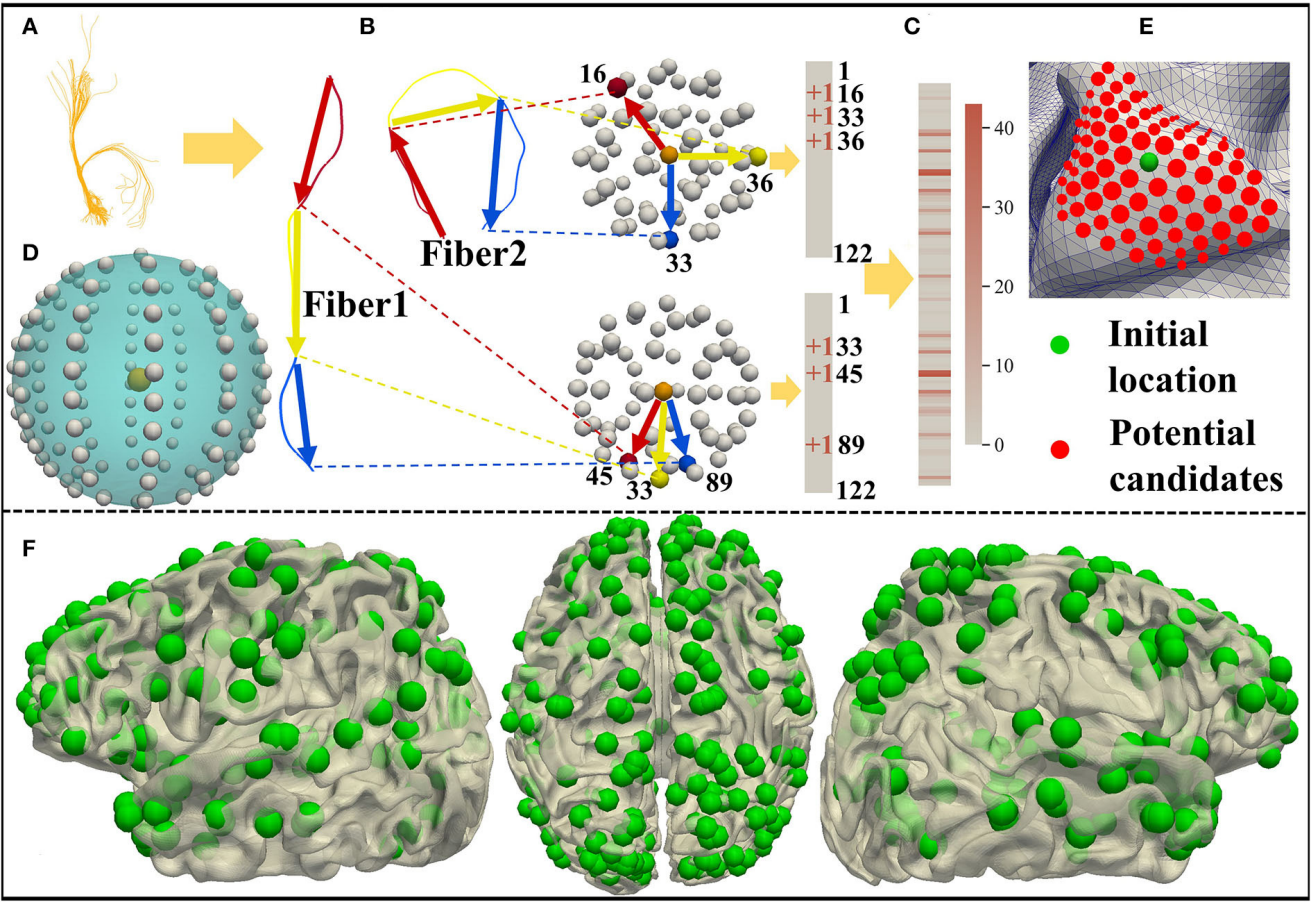
**(A)** A bundle of fibers passing through the DICCCOL landmark. **(B,C)** Trace-map approach. **(D)** Sphere containing 122 regions. **(E)** Initial location and potential candidates of DICCCOL landmark. **(F)** DICCCOL landmarks on one term subject visualized from three perspectives of the left hemisphere, medial view, and right hemisphere.

As shown in Figure 1, we predict 358 DICCCOL landmarks on each subject using the cortical surface and nerve fibers of the brain (Zhu et al., 2013). To be self-contained, the pipeline of calculating the trace-map vector and then optimizing the DICCCOL landmarks on each individual is proposed. Specifically, we use a trace-map approach to describe the shape of nerve fibers. An example of a fiber bundle is visualized in Figure 2A. As shown in Figure 2B, we divide each fiber into three parts evenly, obtain the main direction of each part (from the starting point to the end point), and project the starting point of each part to the center of the sphere and the end point to the sphere. The sphere is divided into 122 equal regions to describe the location of the end point (Chen et al., 2013) (Figure 2D). According to the position of the end point on the sphere, each part of a fiber generates a 122-dimensional vector, and these vectors of a bundle of fibers are accumulated to obtain a representative 122-dimensional vector to describe the shape of the fiber bundle that finally passes through the landmark (Figure 2C). Zhu et al. (2013) provide 10 DICCCOL templates which contain locations of 358 DICCCOL landmarks and corresponding trace-map feature vectors, respectively. When predicting DICCCOL landmarks on a new subject, we first register the cortical surface of the subject to a DICCCOL template and then localize the initial location of each DICCCOL landmark based on the registered surface. Second, we choose the landmarks of the initial location of nearby five-rings as potential candidates for optimizing each of the final DICCCOL landmarks (Figure 2E). Further, for optimizing DICCCOL landmark i on a new subject, correlation coefficient of the trace-map vectors between the potential candidates of DICCCOL landmark i and corresponding landmarks on 10 DICCCOL templates is utilized for optimization; thus DICCCOL landmark i can be identified with the highest correlation coefficient among those potential candidates (Zhu et al., 2013). In this way, the corresponding DICCCOL landmark i can be obtained across all the subjects. Additionally, all the DICCCOL landmarks can be identified by repeating such processes for every initial location of each subject. We show DICCCOL landmarks on a term subject in Figure 2F. The advantage of the DICCCOL system is that DICCCOL landmarks can provide consistency and correspondence across subjects. Therefore, DICCCOL system can overcome the huge individual differences among the subjects and provide the same dimension and one-to-one correspondence data for each subject.

##### Generate feature of graph nodes

Features of graph nodes are generated from the functional MRI data. Specifically, we use the functional similarity between DICCCOL landmarks as features of nodes in the graph. Based on the coordinates of DICCCOL landmarks, the functional time series of DICCCOL landmarks are extracted from the rs-fMRI data, resulting in 358 vectors in length of 2,300 for one subject. We calculate the Pearson correlation coefficient of functional time series between pairs of DICCCOL landmarks to obtain a functional similarity matrix $F_{i}\in {\mathbb{R}}^{358\times 358}$ for one subject. To make the matrix more effective and representative, we set the significant threshold *f*_*s*_ to make the matrix much sparser. Then, for all preterm subjects, we extract the elements of the *i*th row and *j*th column of matrix *F* on each individual to obtain a vector *V*_*p*_ of length 43 (number of all preterm subjects) and use the same method to obtain the vector *V*_*t*_ for term subjects. We perform a *t*-test (two-sample) on *V*_*p*_ and *V*_*t*_ under the significant difference threshold *f*_*s*_ in the training set. If there is a significant difference, we keep the element at the corresponding position of the matrix *F*; otherwise, we set it to 0. The new matrices of all the subjects obtained are stacked, and the obtained $f_{F}\in {\mathbb{R}}^{\left(358\times N\right)\times 358}$ is used as an input to the first convolutional layer, where *N* represents the number of subjects.

##### Generate graph edges

Structural representations of the brain are utilized to represent the graph edges. Specifically, we use nerve fiber bundles as edges of the graph. We reconstruct the nerve fiber tracts of the brain from the dMRI data. We count the number of nerve fibers between all DICCCOL landmark pairs and generate a 358^*^358 structural connection matrix, *S*∈ℝ^358 × 358^ for each individual. The value of the node *S*_*ij*_ represents the number of nerve fibers passing through both DICCCOL *i* and DICCCOL *j*. Likewise, we set the threshold, *t*_*s*_ to make the matrix sparse. We set the value above threshold, *t*_*s*_ to 1 in the matrix *S*, and zero in the others to generate the edge matrix, *E*. The main diagonal element of matrix *E* is also 1. Matrix *E* is used as the edge matrix input of the algorithm.

#### Feature extraction

As shown in Figure 1B, the feature extraction module is designed with three graph convolutional layers, and Layer Normalization is used for normalization in the middle of every two layers. Graph convolution is similar to convolution on image, where Fourier transform is used to transform the convolution into the product of the spectral domain. The transformation matrix can then be used as the convolutional kernel. We obtain the feature output *f*_*h*_ of the graph convolutional layer by Equation (1). Convolution operations are performed on both functional and structural networks since structural information is utilized as the edges of the graph and functional information is utilized as the features of the nodes of the graph.


(1) $$\begin{array}{l} \begin{array}{c} f_{h}=Ẽf_{in}W\\ Ẽ={\;\Lambda \;}^{-\frac{1}{2}}\left(E+I\right){\;\Lambda \;}^{-\frac{1}{2}} \end{array} \end{array}$$

where *f*_*in*_ is the input of the graph convolution layer, W is the transformation matrix, Λ is the degree matrix of *E*, and the diagonal matrix of $\Lambda_{ii}=\;\underset{j}{\sum }E_{ij}+\;1$.

#### Graph pooling

In the graph pooling module, we use the SAGPOOL architecture to accomplish graph pooling. In details, we calculate the attention score *Z* of each node according to Equation (2). The node score *Z* represents the potential contribution of the corresponding DICCCOL landmark in the classification. We reserve nodes with higher node scores *Z* to complete the graph pooling process. We concentrate the outputs of the three graph convolutional layers in the feature extraction module together into the graph pooling layer to obtain the pooled graph nodes.


(2) $$\begin{array}{l} \begin{array}{c} \begin{array}{c} f_{c}=\;concatenate\left(f_{1},\;f_{2},f_{3}\right)\\ f_{m}=\;SelfAttentionPooling\left(f_{\text{c}}\right)\\ Z=\sigma \left(Ẽf_{c}W_{s}\right) \end{array}\\ Z_{m}=Z_{index}\\ index=top\left(Z,\left[kN\right]\right) \end{array} \end{array}$$

where *f*_*c*_ is the feature matrix obtained by concatenating the features from three graph convolution layers and *f*_*i*_ is the output of the *i*th convolutional layer. $W_{s}\epsilon {\mathbb{R}}^{F\times 1}$ is a weight matrix, *kϵ*(0, 1] is a hyper-parameter that determines whether the nodes are eliminated or not. In this experiment, *k* is set as 0.2. The value of *Z* is obtained from the convolutional layer with the size of 1 × *N*; according to the value of *Z*, *top*[*kN*] nodes are remained as important nodes, *and top* is a function defined to return the indices of those most important nodes. *Z*_*m*_ is the final top rank nodes that need to be reserved for the further analysis.

#### Graph classification

In the graph classification module, we feed the node feature of different subjects into the graph readout layer to obtain the features of different graphs and use two fully connected layers to classify them. After the graph pooling layer, due to the reduction of graph nodes, the number of features of each node is correspondingly reduced. In the graph readout layer, we compute the mean and maximum of features of each graph nodes and concentrate them together. The fully connected layer outputs a value for each category of preterm birth and full term, and the category with the highest value is used as the classification result. We obtain the graph feature *f*_*G*_ from the graph readout layer by Equation (3).


(3) $$\begin{array}{l} \begin{array}{c} f_{GE}=globalaverage\left(f_{m}\right)\\ f_{GM}=globalmaximum\left(f_{m}\right)\\ f_{G}=concatenate\left(f_{\text{GE}},f_{\text{GM}}\right) \end{array} \end{array}$$

where *f*_*GE*_ and *f*_*GM*_ are graph features obtained after the global average and the global maximum of nodes, *and f*_*G*_ *is obtained by concatenating* *f*_*GE*_ *and* *f*_*GM*_.

### Differentiating preterm and term infant brains *via* DM-GNN

Based on the proposed DM-GNN framework, experiments are designed to differentiate preterm and term infant brains. Totally, 86 infant brains are adopted, including an equal number of preterm and term infants. The ratio of training data and testing data is 4:1 and a 5-fold cross-validation strategy is utilized. It is worth noting that our input is shuffled. That is, the input node feature matrix, *f*_*in*_ and adjacency matrix, *A* are reconstructed based on the sample ordinal numbers in a random order. We use accuracy (ACC), sensitivity (SEN), specificity (SPE), and receiver operator characteristic (ROC)-area under curve (AUC) to measure the performance of the framework. In particular, we combine the results of 5-folds of test data to calculate the value of ROC-AUC.


$$\begin{array}{lll} ACC & = & \frac{TP\;+TN}{TP+FN+TN+FP} \end{array}$$

(4) $$\begin{array}{lll} SEN & = & \frac{TP}{TP+FN} \end{array}$$

$$\begin{array}{lll} SPE & = & \frac{TN}{TN+FP} \end{array}$$

where *TP* is the number of preterm infants judged to be preterm, *FP* is the number of term babies judged to be preterm, *TN* is the number of term babies judged to be full term, and *FN* is the number of preterm babies judged to be full term.

To verify the value of multimodal fusion, we adopt the single modality GNN model as the ablation experiment. Specifically, instead of using functional similarity matrix as the input for the feature of nodes of DM-GNN model, we adopt DICCCOL landmarks as the graph nodes and their structural trace-map vectors to represent the feature of the graph nodes. In this way, the input of the ablation experiment uses structural profiles sorely. The comparison between the proposed DM-GNN and the ablation experiment can reveal the improvement of the multi-modality fusion.

### Analysis of the differentiable brain regions and identification of the biomarkers

In Section Graph pooling, according to the self-attention pooling layer of the SAGPOOL model, we obtain the *Z* scores of all DICCCOL landmarks when training the DM-GNN model, which represents the contribution of DICCCOL landmarks to the classification performance. We normalize the score *Z* on each subject and obtain the mean of the score *Z* of each DICCCOL landmark on preterm subjects, term subjects, and all the subjects as the classification contribution of this DICCCOL landmark. Those DICCCOL landmarks with high contribution extracted according to a certain proportion, are the candidates for the biomarkers to differentiate the preterm and term infant brains.

### Further interpretation of the biomarkers based on the UNC infant cortical surface atlas

To better interpret those identified biomarkers (important DICCCOL landmarks), UNC infant cortical surface atlas (Li et al., 2015; Wang et al., 2019; Wu et al., 2019) is utilized as the template for all the infant brains. The UNC infant cortical surface atlas is a spatiotemporal cortical surface atlas for infant brains, which is the first spatiotemporal (4D) high-definition cortical surface map.

Two schemes are provided here to further interpret the biomarkers based on the UNC infant cortical surface atlas (i.e., registration priority scheme and average priority scheme). For registration priority scheme, we register the important DICCCOL landmarks on each subject obtained in Section Analysis of the differentiable brain regions and identification of the biomarkers to the standard UNC infant cortical surface atlas to obtain the distribution of these nodes over the brain regions. We then investigate the average of these distributions among subjects. For the average priority scheme, we count the mean of the normalized *Z* score of each DICCCOL landmark across all the subjects and select the serial number of the important DICCCOL landmarks. Similarly, we register the corresponding DICCCOL landmarks on each subject to obtain the mean distribution of nodes on brain regions. We compare these two distributions to analyze differences for preterm vs. term infants and obtain most important brain regions. We extract the DICCCOL landmarks in these regions, count the number of nerve fibers passing through the DICCCOL landmarks in different regions, and finally visualize them.

Using the infant cortical functional parcellation map provided by the UNC infant cortical surface atlas, we analyze the potential impact of preterm birth on brain function. We use the parcellation maps for infants at 3, 6, 9, and 12 months provided by the UNC infant cortical surface atlas, with 7 functional partitions for 3 months, 9 partitions for 6 months, and 10 partitions for others. We calculate the mean value of the distribution ratio of important DICCCOL landmarks among the functional partitions over all the subjects.

## Results

We train and test the algorithm on Pytorch (1.8.0) in a Python (version 3.7.12) environment using an NVIDIA Geforce GTX 3090 with 24GB GPU memory. The order of subjects entered in the algorithm is randomly shuffled. We use the adaptive moment estimation (Adam optimizer) in the model and set the learning rate of the model to 0.0003, and the weight decay to 0.002. We use 5-folds cross validation for experiments. We divide the data into 5 sets equally, and each experiment is performed in a total of *5* times. One set of data is used as the test set in each experiment, and the other four sets are used as the training set. All subjects are used as test subjects once. Considering the actual training performance, we fix the training model for 800 epochs.

### Classification performance between preterm and term infants

**Figure 3:**
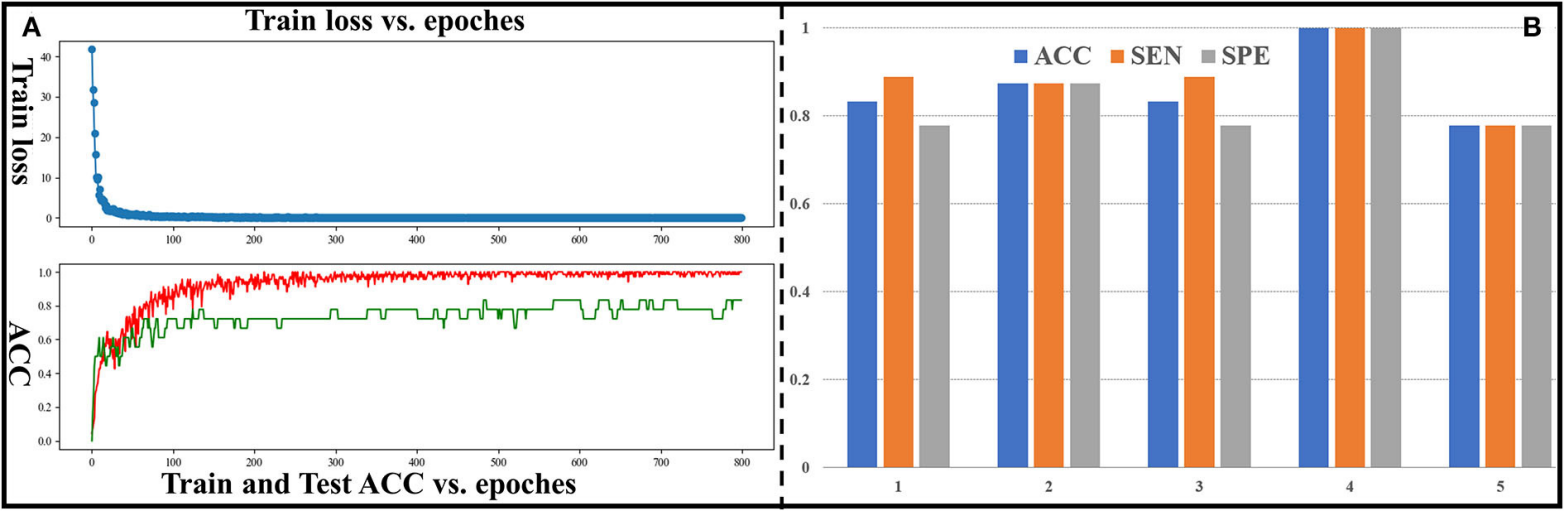
**(A)** Curve of train loss and ACC for the fourth group. The red curve represents ACC of the training set, and the green curve represents ACC of the test set. **(B)** ACC, SEN, and SPE for all groups.

In this section, classification performance of DM-GNN (5-folds cross validation) for binary classification is shown in Figure 3. The curve of training loss and testing ACC for 1-fold is shown in Figure 3A. Mean and standard variation of the 5-folds are 0.864 and 0.084 for ACC, 0.882 and 0.079 for SEN, 0.851 and 0.098 for SPE, and 0.860 for ROC-AUC. ACC, SEN, and SPE of *5*-folds is shown in Figure 3B. As shown in Figure 3B, most of the ACC of 5-folds is higher than 0.80, the SPE and SEN of 3-folds are equal, and the difference between SPE and SEN of the other 2-folds is 0.11. Considering that our dataset is not large, and the test set is lightweight, such fluctuations are acceptable. This shows that the classification performance is stable between 5*-*folds. These results demonstrate the stability and effectiveness of our proposed method and confirm the existing and predictable differences between preterm and term infant brains.

As mentioned in Section Description of DICCCOL-based multi-modality GNN, we conduct supplementary experiments for different values of the number of layers and the number of hidden layer units, which are summarized in Table 1. Three convolutional layers yield the best classification performance and significantly outperforms the others. To determine the number of the hidden units, 5 different scales are chosen: 32, 48, 52, 56, 60, 64, 68, 72, 76, 80, and 96. Their classification performance is reported in Table 2. As shown in Table 2, the effect of the number of hidden units on the results is gradual and 64 hidden units yield the best classification performance. Therefore, the number of the graph convolutional layers is fixed as 3 and the number of the hidden units is set to 64.

**Table 1:** Classification performance of different number of graph convolutional layers.

**Layer number**	**2**	**3**	**4**
ACC	0.719	0.864	0.688
SEN	0.700	0.882	0.789
SPE	0.739	0.851	0.586

**Table 2:** Classification performance of different hidden units.

**Hidden units**	**32**	**48**	**52**	**56**	**60**	**64**	**68**	**72**	**76**	**80**	**96**
ACC	0.640	0.708	0.735	0.768	0.801	0.864	0.815	0.806	0.757	0.769	0.660
SEN	0.575	0.608	0.724	0.825	0.754	0.882	0.835	0.778	0.668	0.817	0.744
SPE	0.706	0.808	0.742	0.728	0.835	0.851	0.801	0.825	0.821	0.722	0.575

As mentioned in Section Differentiating preterm and term infant brains *via* DM-GNN, we conduct ablation experiments using purely structural data as the input to the SAGPool model. The mean ACC of the experiment is 0.758, SEN is 0.769, SPE is 0.747, and ROC-AUC is 0.833. Although the experimental result is not as good as the original experiment, which is around 10% drop, we think this result is reasonable. The reasons are as follows. First, this experiment uses the overall attention (at group level) as the basis of selecting DICCCOL landmarks, while the original experiment uses the attention of each subject. Therefore, our expectations for the results of this experiment are slightly lower than those of the original experiments. Second, parameters are not fine-tuned, such as selecting DICCCCOL with the highest attention 30%; the performance of this experiment still has some space for further improvement. Finally, how to use those biomarkers for the best differentiation are still under investigation; for example, whether generating the significant brain network based on those biomarkers (selected DICCCOL nodes) can bring the best differentiation performance. We will work on those issues in our future work.

### Discriminable biomarkers obtained from proposed method

**Figure 4:**
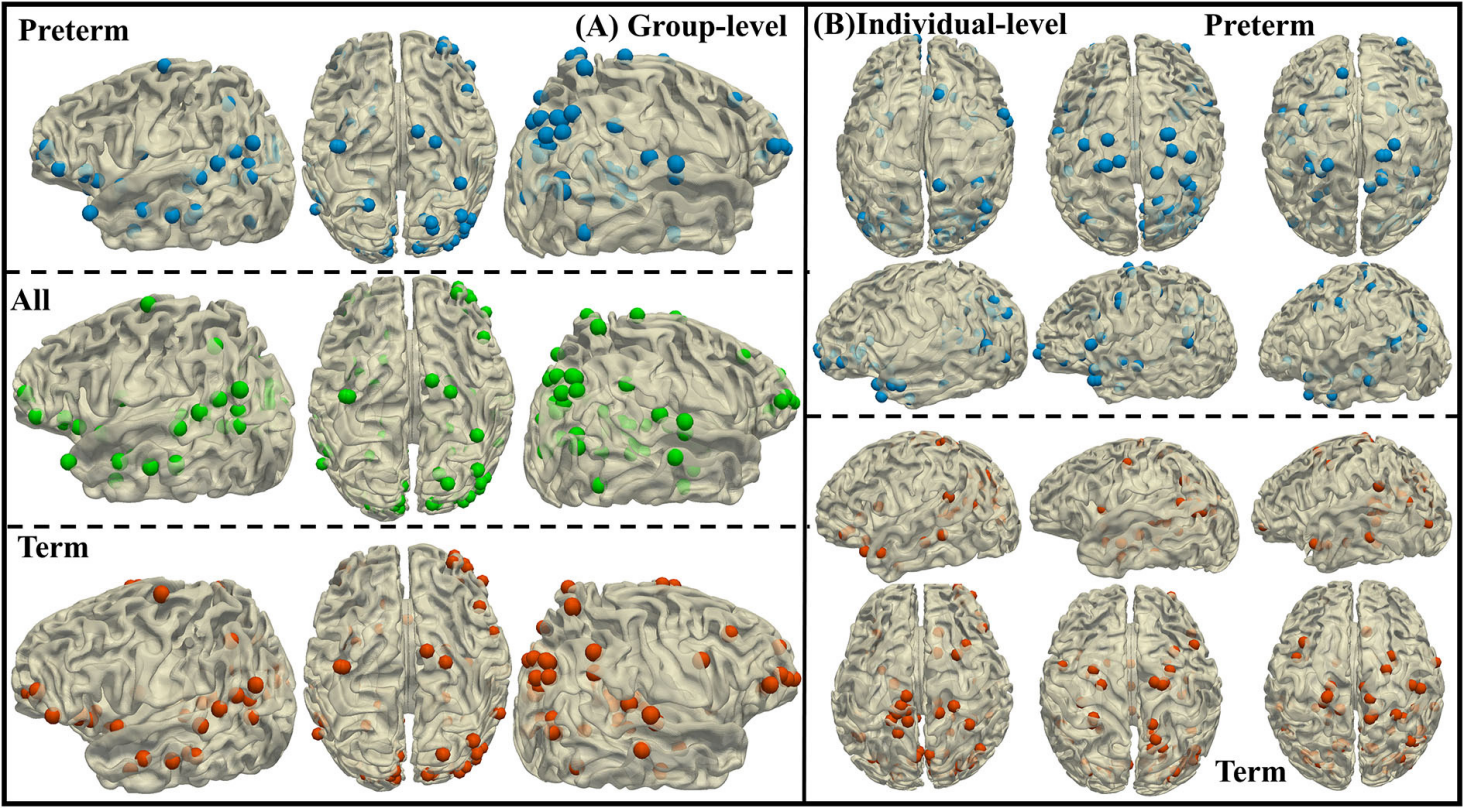
**(A)** Visualize the important DICCCOL landmarks of preterm subjects, term subjects, and all the subjects on a random subject. **(B)** Visualize the important DICCCOL landmarks on preterm and term subjects.

As mentioned in Section Analysis of the differentiable brain regions and identification of the biomarkers, SAGPOOL reveals the distinguishing ability of all the graph nodes. Top 30% important DICCCOL landmarks are retained and shown in Figure 4. For group-level, we obtain group-wise important DICCCOL landmarks from all preterm infants, all term infants, and all individuals separately and visualize on a subject as shown in Figure 4A. For individual-level, we obtain important DICCCOL landmarks from each individual and visualize them in Figure 4B on 3 preterm and term subjects. Interestingly, the overlap of most important DICCCOL nodes between preterm and term subjects is 98/107, and 103/107 between all the subjects and preterm subjects, 102/107 for all the subjects and term subjects. This suggests that most important DICCCOL landmarks, which contribute significantly to the classification with respect to functional and structural representations, are stable between preterm and term subjects, and thus could serve as potential biomarkers of preterm and term infant brains. Due to the huge inter-individual variability, we also evaluate the individual differences of our results. On average, the overlap of important DICCCOL landmarks between a single preterm subject and group-wise ones on all preterm subjects is 68.07 /107 (about 64%), and the corresponding value for term subjects is 69.16/107 (about 65%), suggesting that the important DICCCOL landmarks on the group level are relatively close to those on individual level.

### Investigate biomarkers/regions based on the UNC infant cortical surface atlas

**Figure 5:**
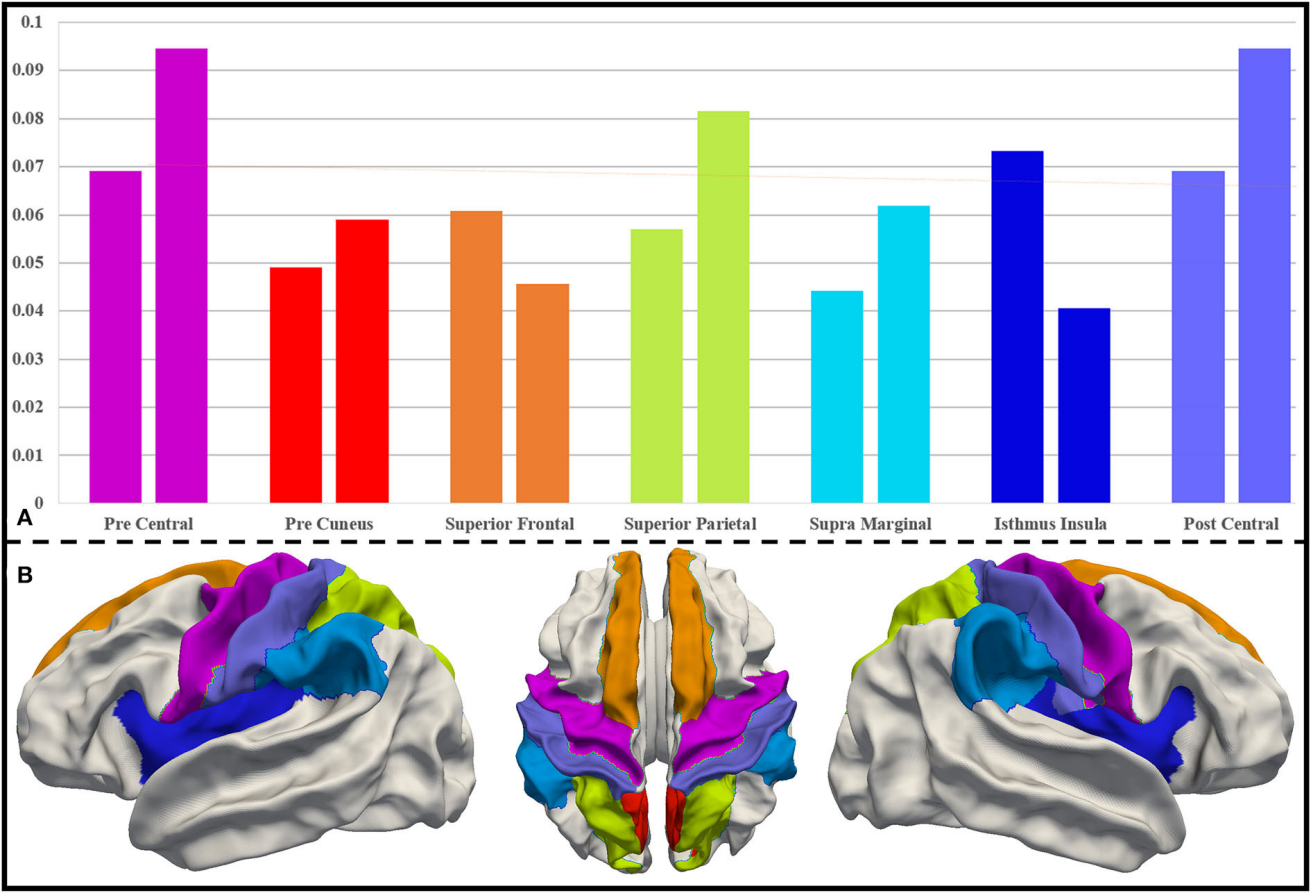
**(A)** Distribution of DICCCOL landmarks in important brain regions for both schemes. For each pair of columns, the left side represents the distribution follow registration priority scheme, and the right side represents the distribution follow average priority scheme. **(B)** Important brain regions on the UNC infant cortical surface atlas, and the color of the brain regions corresponds to that in **(A)**.

As mentioned in Section Further interpretation of the biomarkers based on the UNC infant cortical surface atlas, we extract the most important DICCCOL landmarks using the proposed two schemes at a ratio of top 30%. The region-level distribution of the brain of these important DICCCOL landmarks is obtained after being registered onto the UNC infant cortical surface atlas. We compare the two distributions of two schemes and pick out the brain regions with the highest importance from 36 brain regions, which should be considered as biomarker regions. They are precentral (0.069, 0.095), precuneus (0.049, 0.059), superior frontal (0.061, 0.046), superior parietal (0.057, 0.082), postcentral (0.069, 0.095), supramarginal (0.044, 0.062), and isthmus insula (0.073, 0.041). Their importance level and locations are visualized in Figure 5.

We investigate the abovementioned biomarkers through a literature review, summarized in Table 3. In general, precentral, postcentral, and isthmus insula are more studied and mentioned, and preterm and term infants have greater differences in these regions, which can be considered as more important biomarkers. Other important conclusions from the literature are summarized as follows: (1) Preterm infants have lower axial diffusivity (AD), radial diffusivity (RD), mean diffusivity (MD), and FA in the precentral and postcentral regions, indicating that these regions are less mature than other cortical regions. Conversely, the lower diffusivity (MD, AD, and RD) of isthmus insula indicates a higher maturity. (2) The precentral, postcentral, and isthmus insula regions contain brain hubs, and the precentral, precuneus, and post central regions can predict brain age in preterm infants using functional data. (3) Precentral, precuneus, and superior frontal are identified as higher-order rich-clubs, and superior parietal, supramarginal, and isthmus insula contain rich-club nodes. These reports demonstrate that our method to find biomarkers is effective. On this basis, other less concerned regions, including precuneus and supramarginal, could also deserve a further attention, even though fewer reports on them are available.

**Table 3:** The literature review of the proposed brain biomarker regions.

**Region**	**References**	**Description**
Pre central	1. Ouyang et al., 2019	1. Mean kurtosis (MK) is low at 33 weeks and decrease significantly at 33–40 weeks. (Preterm and structure)
	2. Bouyssi-Kobar et al., 2018	2. The values of AD, RD, MD, and FA are significantly higher. (Preterm compared with term and structure)
	3. Yu et al., 2016	3. FA values are higher at 20 weeks and lower at 35 weeks. (Structure)
	4. Fouladivanda et al., 2021	4. This region is identified as a higher-order rich-club. (Term, structure, and function)
	5. Xu et al., 2019	5. The variance of FC of preterm around 40 weeks is significantly lower than preterm around 34 weeks. (Preterm and function)
	6. Cao et al., 2017	6. This region is a functional hub and can be used brain maturity prediction. (Preterm and function)
Pre cuneus	1. Sa de Almeida et al., 2021	1. The connection strength of preterm subjects is lower than that of term subjects. (Preterm compared with term and structure)
	2. Fouladivanda et al., 2021	2. This region is identified as a higher-order rich-club. (Term, structure, and function)
	3. Cao et al., 2017	3. This region can be used brain maturity prediction. (Preterm and function)
Superior frontal	1. Ouyang et al., 2019	1. MK decreased significantly and is low at 40 weeks. (Preterm and structure)
	2. Ball et al., 2013	2. High MD values for cluster containing in this region. (Preterm compared with other clusters and structure)
	3. Fouladivanda et al., 2021	3. This region is identified as a higher-order rich-club. (Term, structure, and function)
Superior parietal	1. Ball et al., 2013	1. It has fast decreasing FA value (preterm at 27–38 weeks) and high MD value for cluster containing this region. (Preterm and structure)
	2. Fouladivanda et al., 2021	2. This region contains rich-club nodes. (Term, structure, and function)
	3. Stoecklein et al., 2020	3. The FC of it show significant differences from adults. (Preterm and function)
Post central	1. Ouyang et al., 2019	1. MK is low at 33 weeks and decreased significantly. (Preterm and structure)
	2. Bouyssi-Kobar et al., 2018	2. The values of AD, RD, MD, and FA are significantly higher. (Preterm compared with term and structure)
	3. Yu et al., 2016	3. FA is high at 20 weeks and low at 35 weeks. (Structure)
	4. Xu et al., 2019	4. The variance of FC of preterm around 40 weeks is significantly lower than preterm around 34 weeks. (Preterm and function)
	5. Cao et al., 2017	5. This region is a functional hub and can be used brain maturity prediction. (Preterm and function)
Supra marginal	1. Ball et al., 2013	1. The value of FA decreases significantly during 28–38 weeks. (Preterm and structure)
	2. Fouladivanda et al., 2021	2. This region contains rich-club nodes. (Term, structure, and function)
	3. Stoecklein et al., 2020	3. The FC of it show significant differences from adults. (Preterm and function)
Isthmus insula	1. Ouyang et al., 2019	1. The values of MK and FA decreased slowly. (Preterm and structure)
	2. Bouyssi-Kobar et al., 2018	2. The lower diffusion rates (MD, AD, RD) in this region. (Preterm compared with term and structure)
	3. Sa de Almeida et al., 2021	3. In both preterm and term, it contains brain hubs. (Both preterm and term and structure)
	4. Fouladivanda et al., 2021	4. This region contains rich-club nodes. (Term, structure, and function)

**Figure 6:**
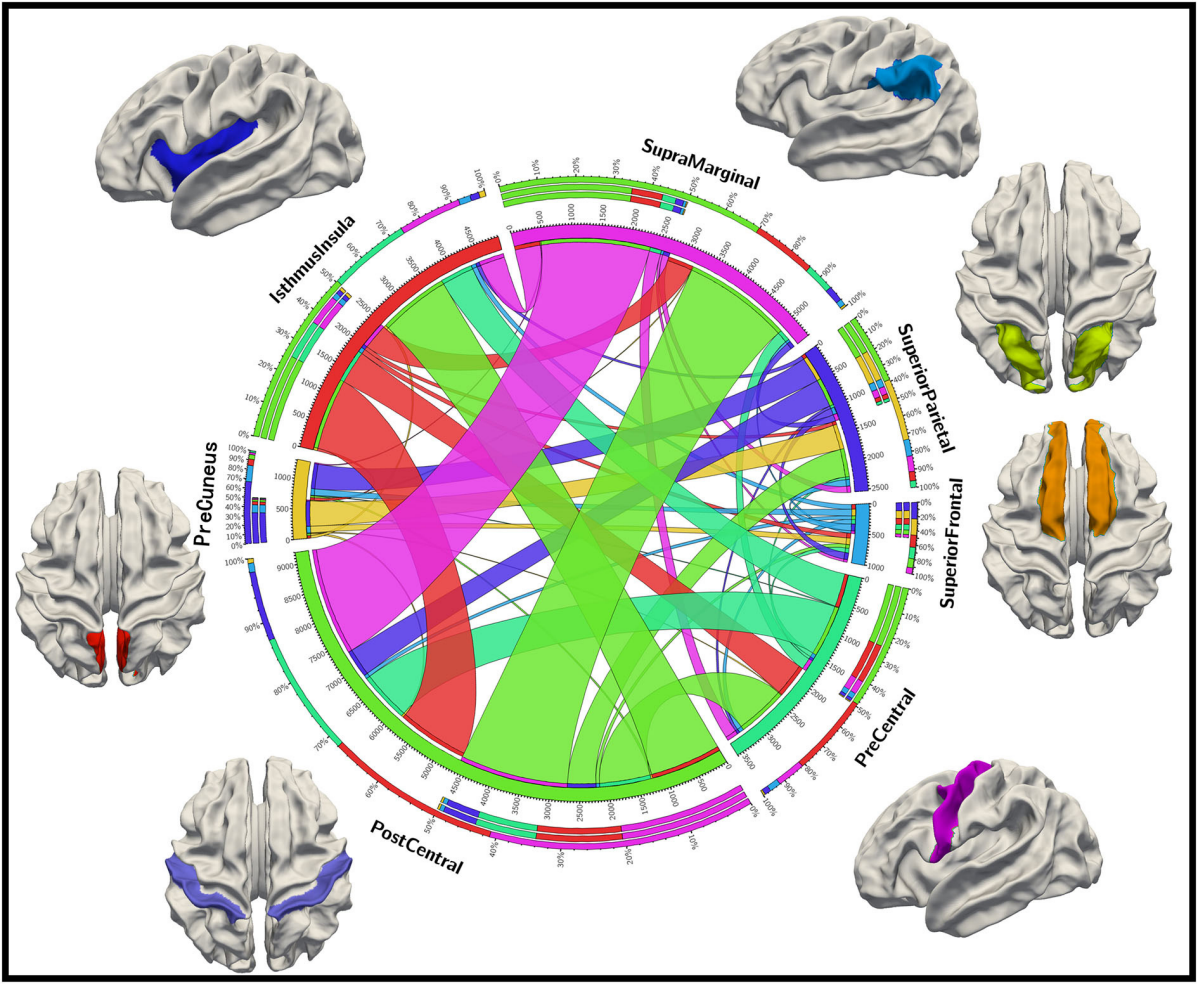
Structural connection patterns among important brain regions.

To further analyze the relationship between these identified brain biomarker regions, we investigate the structural connectivity of the biomarkers (important DICCCOL landmarks) in those brain regions. As shown in Figure 6, postcentral, isthmus insula, and supramarginal have much more connections to other regions, suggestive of the greater importance of those brain regions as the “connector” of brain regions. In contrast, superior frontal and precuneus are less connected to other regions. Also, the connections among postcentral, isthmus insula, and supramarginal are strong, while postcentral, superior frontal, and isthmus insula have a relatively balanced proportion of connections among each other.

We also use longitudinal UNC infant cortical surface atlas to further study the brain functions of those identified brain biomarker regions. After the mapping between our identified brain biomarker regions and UNC infant cortical surface atlas at different time points are done, we obtain the functional networks with the proportion of the biomarkers participated at different time points (Table 4). It is worth noting that certain brain functions have not developed at early months, so some elements in Table 4 is not applicable. It is found that biomarkers are largely involved in the functional networks of central visual, anterior default mode, and superior temporal lobe, suggesting that those brain functions may include huge differences between preterm and term infant brains.

**Table 4:** Functional networks with the proportion of biomarkers participated at different time points.

**Function networks**	**Hand sensory motor**	**Central visual**	**Anterior default mode**	**Anterior frontoparietal**	**Dorsal attention**
3 months	0.023	0.211	0.197	0.150	-
6 months	0.093	0.190	0.201	0.130	0.033
9 months	0.091	0.147	0.157	0.147	0.045
12 months	0.099	0.162	0.169	0.133	0.045
**Function networks**	**Mouth sensory motor**	**Peripheral visual**	**Posterior default mode**	**Posterior frontoparietal**	**Superior temporal**
3 months	0.120	-	-	0.067	0.232
6 months	0.069	-	0.082	0.058	0.145
9 months	0.057	0.032	0.048	0.053	0.223
12 months	0.069	0.030	0.061	0.056	0.177

## Conclusion and discussion

In this work, a novel DICCCOL-based multi-modality GNN framework is proposed to differentiate preterm and term infant brains and characterize the corresponding biomarkers. Classification accuracy is 86.4% and 7 important brain regions with 107 landmarks are identified as the biomarkers. Our experiment results demonstrate the effectiveness of the proposed framework to differentiate the brain disorders or diseases, as well as the validity of the identified biomarkers. Our results shed the new insight that DM-GNN framework owns the superiority for differentiating brain functional states and identifying the biomarkers. Particularly, utilizing both functional and structural profiles, the DICCCOL system initializes the graph nodes with correspondence but retains the individual differences. As so, multi-modality GNN can better capture the brain functional states and extract meaningful features.

### Connectome-level study from the functional and structural perspective

In recent years, research on preterm birth has gradually shifted from volume-based analysis to connectome-level analysis and we summarize some related studies. With regard to volume-based analysis, more specifically, quantitative MRI studies can be used to explore both WM and GM abnormalities in preterm birth brains. Besides, many studies reported that volume reductions have been described in the hippocampus (Nosarti et al., 2002; Cheong et al., 2013), caudate nucleus (Abernethy et al., 2002; Nosarti et al., 2008), thalamus (Giménez et al., 2006; Cheong et al., 2013), corpus callosum (Nosarti et al., 2004; Narberhaus et al., 2008; Taylor et al., 2011), and cerebellum (Allin et al., 2001; Taylor et al., 2011). Additionally, many related works focused on using voxel-based morphometry to explore the widespread GM and WM alterations of preterm birth, especially in frontal and temporal lobes, which mediated cognitive impairment (Nosarti et al., 2008). Diffusion tensor imaging (DTI) has also been adopted to characterize WM microstructure in the developing brain (Allin et al., 2011).

Studies are proposed to explore the functional or structural alterations at the connectome-level. For example, preterm infants with moderate to severe WM injuries were found to show greater loss of connectivity than very preterm infants without WM injuries and term-born infants (Smyser et al., 2013). Reductions in the functional connectivity between resting state networks (RSNs) had been reported to persist throughout the early childhood (Damaraju et al., 2010). Wehrle et al. (2018), proposed that assessing the functional connectivity of the resting brain should provide valuable insight into underlying mechanisms of impaired cognitive development after preterm birth. Further, a series of machine learning algorithms had been proposed to better characterize the alterations on the connectome-level, e.g., voxel-wise statistical analysis of the diffusion data was performed using tract-based spatial statistics (TBSS, part of FSL) (Eikenes et al., 2011); an artificial neural network (ANN) framework for early prediction of cognitive deficits in very preterm infants based on fMRI connectome data (He et al., 2018); a deep learning convolutional neural network had been proposed to identify preterm infants at the risk of a later motor impairment and to identify brain regions with predictors of adverse outcome (Saha et al., 2020).

Based on these connectome-level analyses, a variety of different opinions and observations about the alterations caused by the preterm birth are obtained. On the one hand, studies on the structural connectivity proposed that the brains of preterm infant show increased modularity, weakened rich-club connectivity, and diminished global efficiency compared to term infants, suggesting a delayed transition from a local architecture, i.e., focused on short-range connections, to a more distributed architecture with efficient long-range connections (Sa de Almeida et al., 2021). On the other hand, studies on the functional connectivity had largely focused on connections involved in language and attention, which reported profound alterations in the functional connectivity within and between language areas and language-related areas, and other parts of the brain, such as visual attention and working memory areas (Finke et al., 2015). However, dissenting opinion was also found, e.g., two types of relationship are possible: (i) the more attention is impaired, the more intrinsic connectivity is changed from that of healthy controls, reflecting detrimental effects of preterm birth; (ii) the less attention is impaired, the more intrinsic connectivity is changed from that of healthy controls, reflecting compensatory response on effects of preterm birth (Finke et al., 2015). Eikenes et al. (2011), also stated that structural and functional alterations were not always consistent, and different trajectories in brain connectivity could exist. Therefore, the joint analysis of function and structure is extremely important.

### Experiment of more fine-grained division of preterm birth data

Preterm birth is a dynamic process that also varies among preterm infants of different gestational ages. We try to do a more fine-grained division of the preterm birth data. We further divide the data of preterm infants into three subcategories: “Group 1”: 18 infants younger than 32 weeks of gestational age; “Group 2”: 12 infants between 32 and 35 weeks of gestational age; “Group 3”: 13 infants older than 35 weeks of gestational age. Three binary classification experiments are then designed; they are “Group 1” vs. term infants, “Group 2” vs. term infants, and “Group 3” vs. term infants. To keep the balance of the input data, for each binary classification, the number of the term infant brains we used is exactly the same as the preterm infant brains. The results are shown in Table 5.

**Table 5:** Classification performance of term and preterm infants in fine-grained division.

	**Group 1 vs. term**	**Group 2 vs. term**	**Group 3 vs. term**
ACC	0.800	0.767	0.733
SEN	0.767	0.733	0.767
SPE	0.833	0.800	0.700
ROC-AUC	0.875	0.806	0.667

The ACC for classification between Group 1 and term infants is 0.800, and the ROC-AUC is 0.875, which is the best among the three groups of experiments. The ACC for classification between Group 2 and term is 0.767, and the ROC-AUC is 0.806, which is slightly worse than the previous experiments. The ACC for classification between Group 3 and term is 0.733, and the ROC-AUC is 0.667. These results suggest that the differences between preterm and term infant brains are much more significant in preterm infants at younger gestational age. The overall classification performances of these three experiments are lower than that of the original experiments, due to the relatively small number of subjects used in the experiments resulting in a low generalization ability of the features learned by the model. This is why we prefer not to split the preterm data into a finer granularity. Our follow-up research will pay more attention on this issue and try to include more available data into consideration.

### Discussion on the effectiveness of the DICCCOL system

**Figure 7:**
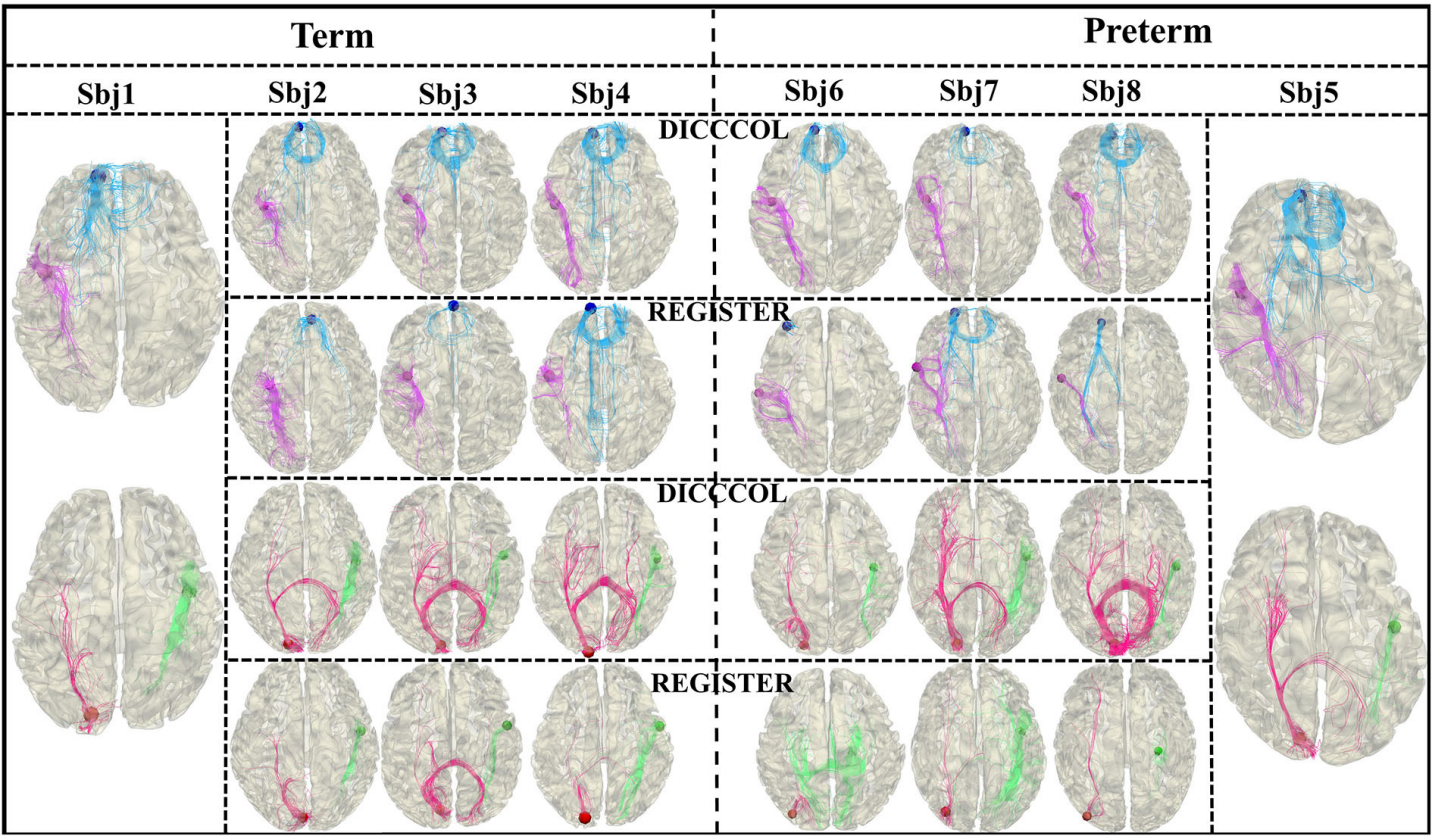
Fiber shapes of DICCCOL landmarks and registered landmarks.

Since both functional information and structural information are carried by the DICCCOL landmarks that construct the graph nodes, the functional and structural consistency of DICCCOL landmarks across the subjects is crucial. Two experiments are designed to demonstrate the effectiveness of functional and structural correspondence of DICCCOL across the subjects. For the structural perspective, we randomly select 4 preterm subjects as category 1 and four term subjects as category 2, respectively. For each category, we select one subject as a template, and register the DICCCOL landmarks of the template to the other three subjects. As shown in Figure 7, for each category, we visualize the shape of fibers passing through 4 DICCCOL landmarks and registered landmarks. It is clear that the shapes of fibers passing through the DICCCOL landmarks are much more similar, illustrating the advantages of the DICCCOL system on structural consistency over traditional registration algorithm.

**Figure 8:**
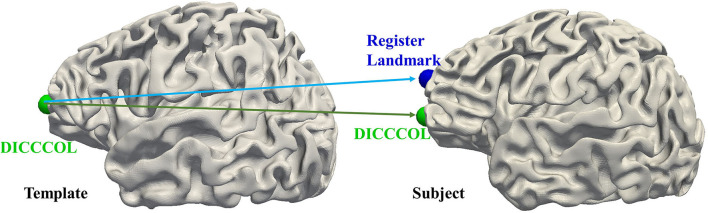
Illustration of functional consistency between DICCCOL landmark and registered landmark.

For the functional perspective, to use the task fMRI data, we use the Human Connectome Project (HCP) S900 dataset (Van Essen et al., 2013) for the experiment. We select 86 subjects to conduct experiments using their fMRI data for 7 tasks including emotion, gambling, language, motor, relational, social, and working memory. We randomly select one subject as a template and register its DICCCOL landmarks to the other 85 subjects as registered landmarks. Two functional similarities are calculated: (1) between the DICCCOL landmarks on the template and other subjects; (2) the DICCCOL landmarks on the template and the registered landmarks on other subjects (Figure 8). The functional consistency of DICCCOL landmarks is demonstrated by comparing the functional similarities. The functional similarity is represented by the similarity of the functional signals and similarity matrices. The results are shown in Table 6. The functional signal similarity between the two DICCCOL landmarks in Figure 8 is 0.3418, and the functional signal similarity between the DICCCOL landmark on the template and the registered landmark is 0.3027, which is lower than that between the DICCCOL landmarks. As shown in Table 6, similarities between DICCCOL landmarks are slightly higher in six functions than the results of the registration, and equal in the other one. Overall, it shows that the function between DICCCOL landmarks is more stable than using the traditional registration method.

**Table 6:** Similarity comparison between DICCCOL-based and traditional registration method.

**Landmark type**	**Similarity type**	**Emotion**	**Gambling**	**Language**	**Motor**	**Relational**	**Social**	**Working memory**
DICCCOL	Signals	0.0041	0.0116	0.0083	0.0139	0.0149	0.0242	0.0158
	Similarity matrix	0.1853	0.2689	0.2415	0.2391	0.2206	0.2373	0.1866
Registered	Signal	0.0041	0.0085	0.0053	0.0120	0.0117	0.0194	0.0125
	Similarity matrix	0.1853	0.2647	0.2396	0.2355	0.2174	0.2314	0.1835

In general, we are clear about the performance and limitations of DM-GNN. It is worth noting that our proposed DM-GNN is not only designed for the study of preterm and term infant brains, but also established as a standard framework which can be effectively applied to other brain disorders, such as Alzheimer (Parisot et al., 2018), Schizophrenia (Du et al., 2021), and Autism (Du et al., 2021). In the future, we will continuously improve the DM-GNN framework, including the architecture of GNN and the integration of the multi-modality representations.

## Data availability statement

Publicly available datasets were analyzed in this study. This data can be found here: http://www.developingconnectome.org/.

## Ethics statement

The studies involving human participants were reviewed and approved by UK National Research Ethics Authority. Written informed consent to participate in this study was provided by the participants' legal guardian/next of kin.

## Author contributions

SZ, TZ, and RW: conception and design. SZ: analysis and interpretation. ZH, XH, and YZ: data preprocessing. SZ, RW, YK, JWu, and JWa: writing the manuscript. SZ, RW, TZ, and HG: critical revision of the manuscript. SZ and TZ: overall responsibility. All authors contributed to the article and approved the submitted version.

## Funding

SZ was supported by the National Natural Science Foundation of China 62006194; the Fundamental Research Funds for the Central Universities (Grant No. 3102019QD005); High-Level Researcher Start-up Projects (Grant No. 06100-22GH0202178). The National Natural Science Foundation of China (31971288, U1801265) supported TZ.

## Conflict of interest

The authors declare that the research was conducted in the absence of any commercial or financial relationships that could be construed as a potential conflict of interest.

## Publisher's note

All claims expressed in this article are solely those of the authors and do not necessarily represent those of their affiliated organizations, or those of the publisher, the editors and the reviewers. Any product that may be evaluated in this article, or claim that may be made by its manufacturer, is not guaranteed or endorsed by the publisher.
